# Multiple intrahepatic pancreatic pseudocyst (MIHPPs): an overlooked and misdiagnosed entity

**Published:** 2019

**Authors:** Sagar Tomar, Rohini Gupta Ghasi, Juhi Agarwal

**Affiliations:** *Department of Radiodiagnosis and Imaging, Safdarjung Hospital, Vardhman Mahavir Medical College, New Delhi, India *

**Keywords:** pancreatitis, pseudocysts, hepatic cyst, intrahepatic pseudocysts, multidetector computed tomography

## Abstract

Pancreatitis and pseudo-pancreatic cysts are frequently encountered entities; however, intrahepatic pseudocysts presenting as large number of liver cysts with absence of overt signs of pancreatitis has never been reported in literature. Here, we report an interesting case of multiple intrahepatic pancreatic pseudocysts (MIHPPs), a challenging diagnosis to be kept in mind while dealing with complex cystic lesions of liver. Pseudocysts are common complication of pancreatitis, often these are located within the vicinity of the pancreas in the lesser sac and the retroperitoneum. Extra pancreatic location of these cysts within the liver is a diagnosis often missed, with only 50 odd cases reported in literature till date. Most of these reported cases are either subcapsular in location or limited in number to one or two lesions. Although rare, possibility of MIHPPs is an important diagnosis that should be kept in mind while considering list of differentials for complex cystic lesions of the liver even in the absence of overt signs of pancreatitis.

## Introduction

 Pseudocyst formation is a very common complication of acute pancreatitis. Most pseudocysts are located around the pancreatic gland, but they have also been described at a site distant from the pancreas; from the mediastinum to the scrotum, as the fluid dissects through tissue planes (1, 2). A rare location for these pseudocysts is the liver with about 54 cases in literature since the 1970. In majority of the cases, the cyst is single and a maximum of four cysts in a patient have been reported till date (3). Correct diagnosis is often not difficult in presence of acute pancreatitis or there is direct extension of the fluid along the hepato-gastric ligament; however, it requires high index of suspicion in the absence of signs of pancreatitis and atypical ultrasound appearance. Often these lesions are overlooked and misdiagnosed as multiple hepatic abscesses, hydatid cyst or even multiple metastases in older individuals which significantly hampers the management of these patients. Interest in the presented case arises from not only the fact of rarity of the location of the lesion but also on the atypical imaging appearance of the lesion. 

## Case report

A 68-year-old male with no past history of hepato-biliary or pancreatic diseases came to the emergency room with non-radiating right upper abdominal pain since 10 days. He was chronic alcoholic since 15 years. Physical examination revealed moderate right hypochondrial pain and tenderness. No evidence of jaundice was present. The bowel sounds were normal. 

Abdominal ultrasound demonstrated multiple intrahepatic well defined lesions of variable size involving both lobes of liver with few appearing almost solid while few of them solid –cystic appearance with low level internal echoes and debris. No color flow was noted in the solid component. The liver was also enlarged in size measuring approximately 17cm. Multiple calculi were also seen in gallbladder. The pancreas appeared normal with no evidence of intra-peritoneal free fluid. Initial diagnosis of multiple hepatic abscess or hydatid cyst was conveyed to the referring physician. However, in absence of clinical history of fever and classical US findings of hydatid cyst, further evaluation with CECT abdomen was suggested.

**Figure 1:**
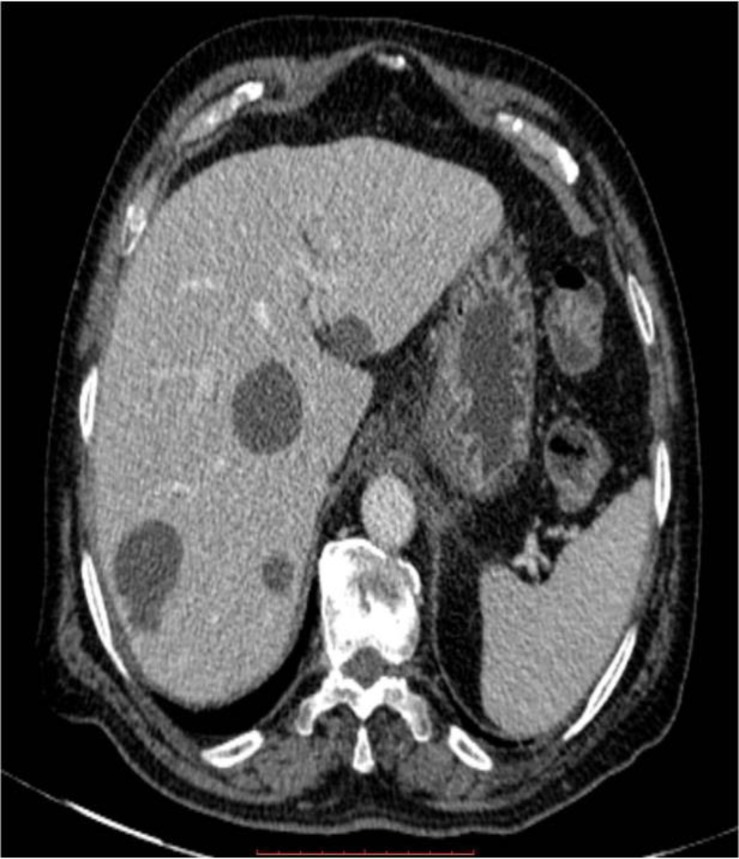
Axial CT section demonstrates multiple non enhancing cystic lesions scattered in both right and left lobes of liver

**Figure 2:**
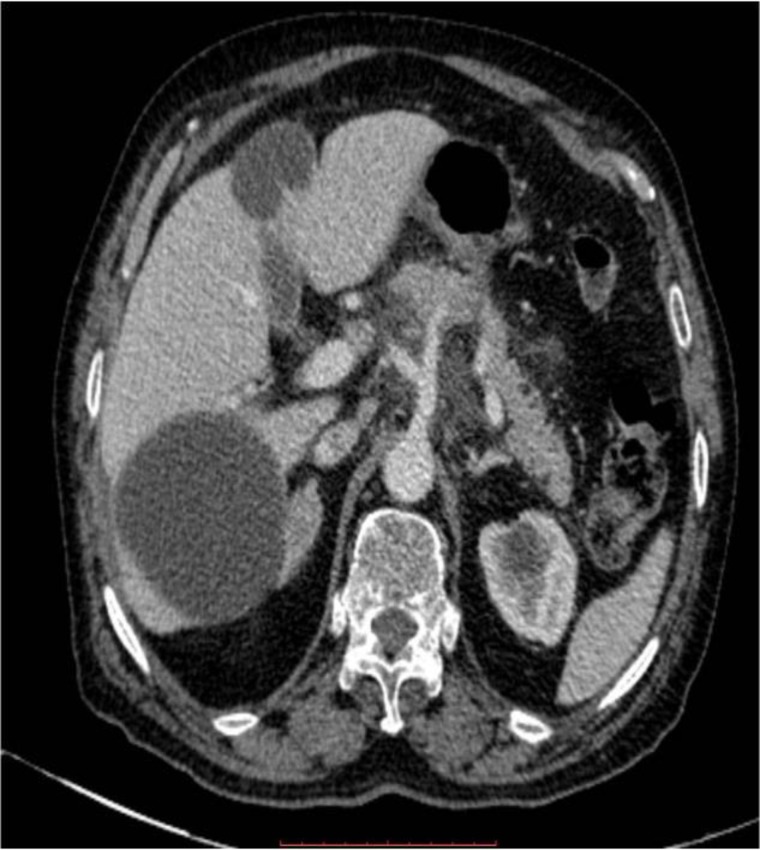
Axial contrast enhanced computed tomography reveals two round well defined homogenous hypodense lesions with thin imperceptible walls in segment V and VI of liver. Mild peripancreatic fat stranding is also present

**Figure 3:**
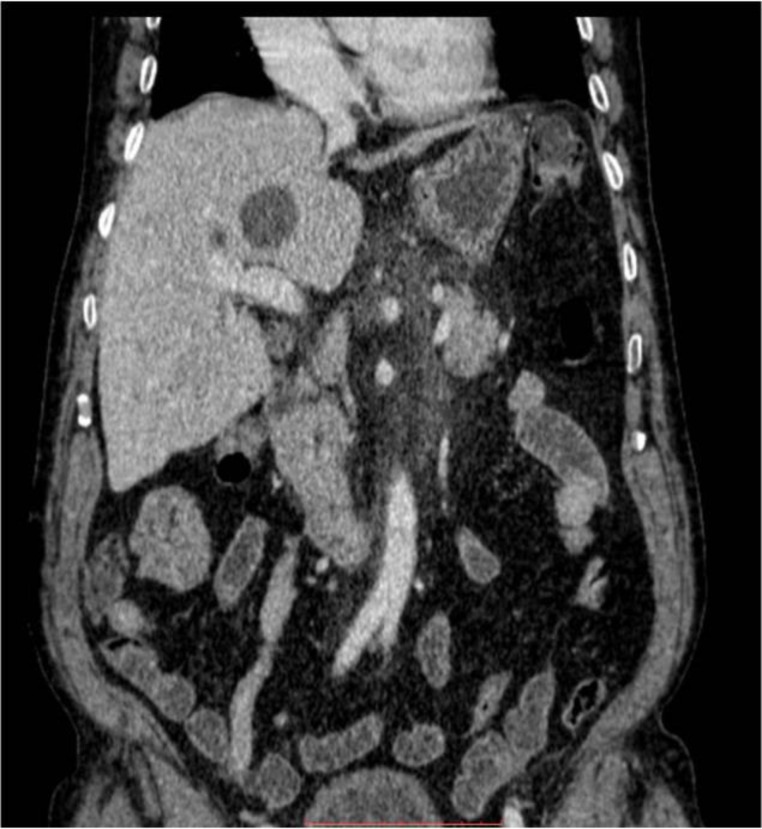
A small round well defined lesion is seen in left lobe of liver with mild to moderate peripancreatic fat standing. No free fluid is seen in the abdominal cavity

 A computed tomography (CT) scan revealed multiple variable sized well-defined, homogeneous, fluid attenuating lesions involving both lobes of liver (Figure 1 and 2). A total of eight cysts were present. There was mild fat stranding in the peripancreatic planes (Figure 3).

 Laboratory investigations revealed 13,500 white blood cells/mm3 (75% neutrophils), normal hemoglobin, 870 IU/L serum amylase (normal value < 115 IU/L), 920 IU/L serum lipase (normal value < 190 IU/L), 320 U/L AST (normal value < 37), 276U/L ALT (normal value < 41) and 251mU/mL alkaline phosphatase (normal value < 300).

Once the echinococcal antigen test was negative, US guided aspiration of fluid was done from one of the cysts. The aspirate revealed a clear fluid with raised level of 1222 U/L amylase and 6000 U/L lipase thus confirming the diagnosis of MIHPPs. The larger cysts were aspirated under US guidance while the smaller cysts were managed conservatively.

## Discussion

Pancreatic pseudocysts, a well-known complication of pancreatitis has virtually been described in all organs of the body. However, intrahepatic location of pseudocyst has rarely been identified (4).

Intrahepatic pseudocysts are usually single and most commonly involve the left lobe of liver, but multiple intrahepatic pseudocysts (MIHPPs) involving both lobes of liver have also been described (5-6).

MIHPPs can be subcapsular or intraparenchymal in location. Two pathophysiological mechanisms have been proposed to explain formation of intrahepatic pseudocysts (7-10). According to first mechanism, there is accumulation of pancreatic juice in peripancreatic and pararenal areas, from where the fluid penetrates through the posterior layer of parietal peritoneum to reach lesser sac. The collection then tracks along lesser omentum and gastrohepatic ligament to reach the left lobe of liver. The leaked fluid dissects along the liver capsule leading to formation of subcapsular collections. The second mechanism proposes the propagation of pancreatic juice from the head of pancreas to the porta hepatis along the hepatoduodenal ligament resulting in formation of multiple intraparenchymal collections. The anatomic location of the case presented here seems to be better described by the second theory (7-10).

Point in contention in our case is unusual appearance of the lesion on US, with lesion appearing as solid and solid - cystic on the background of normal looking pancreas causing diagnostic dilemma at initial evaluation and prompting further imaging work up of the lesion. Such a large number of IHPPs in a single case have never been previously described in literature.

This atypical US appearance of the pseudocysts may be contributed due to debris making the US diagnosis of these lesions difficult. In acute settings, they may appear hyperdense on NCCT scans due to hemorrhage and debris within.

Diagnosis of intrahepatic pseudocyst is a diagnostic challenge as it is not even considered in the differentials for cystic liver lesions in absence of pancreatic pathology. Definite diagnosis of MIHPPs can only be made if aspirate from the lesions reveal elevated amylase/lipase or direct communication can be demonstrated between intrahepatic pseudocysts and peripancreatic collection. ERCP also helps in confirming the diagnosis by demonstrating disrupted pancreatic duct with accumulation of contrast in intrahepatic collection.

Management of intrahepatic pseudocyst tends to differ from pancreatic pseudocyst. No definitive guidelines have been provided regarding the management however USG guided percutaneous drainage is preferred over other options such as surgical resection or transpapillary stenting. 

Possibility of intrahepatic pseudocyst should always be kept in mind in case of acute or chronic pancreatitis presenting with atypical fluid collection in liver. Even in patients without overt signs of pancreatitis, MIHHPs should be kept in the list of differentials for multiple complex cystic lesions of liver, as the patient might have remote history of pancreatitis with complications manifesting at the time of presentation. 

However, confirmatory diagnosis can only be arrived at on demonstration of raised amylase levels in fluid aspirate or direct communication between cyst and peripancreatic fluid collection or pancreatic duct.

## Conflict of interests

The authors declare that they have no conflict of interest.
